# A periodized winter training block enhances anaerobic capacity in elite female rowers during 30-second maximal rowing

**DOI:** 10.3389/fphys.2025.1705448

**Published:** 2026-01-08

**Authors:** Xiaofang Liu, Yuquan Ding, Zhigang Gong, Pengcheng Guo

**Affiliations:** Key Lab of Aquatic Sports Training Monitoring and Intervention of General Administration of Sport of China, Faculty of Physical Education, Jiangxi Normal University, Nanchang, China

**Keywords:** anaerobic capacity, energy expenditure, energy metabolism, rowing, winter training

## Abstract

**Objective:**

In competitive rowing, the winter training phase is a conventional periodized block focused on foundational physiological development through land-based strength and power training. The primary aim of this phase is to enhance athletes’ force-generating capabilities and metabolic resilience. This study investigated the effects of such a 16-week periodized winter training block on anaerobic capacity and energy expenditure during a 30-s all-out rowing test in elite female rowers.

**Methods:**

Five elite female rowers (age: 20.0 ± 2.5 years; BMI: 20.8 ± 0.3 kg/m^2^; training experience: 4.8 ± 1.6 years) underwent pre- and post-training assessments. The winter training program consisted of three sequential phases focusing on aerobic endurance, anaerobic threshold, and race-pace intervals, with 5-6 sessions per week. Testing was conducted on the same day in the following order: (1) a 30-s all-out rowing test to assess anaerobic power output and metabolism; and (2) an incremental rowing test to exhaustion performed after a 10-min recovery, to establish individual oxygen uptake-power relationships for the maximal accumulated oxygen deficit (MAOD) method and to confirm maximal oxygen uptake (VO_2_max).

**Results:**

Following winter training, significant improvements were observed during the 30-s all-out rowing test in mean power output (+14.1%), anaerobic energy contribution (+22.0%), and total energy expenditure (+12.1%), alongside a reduction in aerobic contribution (−16.9%) and oxygen uptake (p < 0.05). Peak post-test blood lactate concentration also increased significantly (p < 0.05). No changes were detected in body mass or energy utilization efficiency (p > 0.05). The incremental test confirmed that V̇O_2_max was maintained post-training (p > 0.05), ensuring that the calculated MAOD reflected true anaerobic adaptations.

**Conclusion:**

Winter training markedly enhanced anaerobic capacity in elite female rowers, facilitated by a pronounced metabolic shift toward anaerobic pathways. Despite increased total energy expenditure, energy efficiency remained unchanged, suggesting improvements were driven primarily by physiological rather than technical adaptations. These findings underscore the importance of anaerobic development in competitive rowing performance.

## 1 Introduction

Rowing has been an Olympic sport since 1900, with competition distances undergoing several modifications. The men’s event was standardized to 2000 m in 1960, while the women’s event—introduced in 1976—adopted the same distance in 1988 (World Rowing Federation). Modern 2000-m rowing races are typically completed in 5.5–8 min and are characterized by distinct energetic demands: 65%–75% reliance on aerobic metabolism and 25%–35% contribution from anaerobic pathways (Schünemann et al., 2023; Zi, 2015). Crucially, during the initial start phase (0–250 m) and final sprint (last 250 m), athletes must generate maximal power within brief durations, where anaerobic capacity (encompassing the phosphagen and glycolytic systems) becomes a decisive performance determinant (Garland, 2005; Schünemann et al., 2023).

Contemporary pacing strategies typically exhibit a parabolic (U-shaped) trajectory (Kleshnev and Nolte, 2001), featuring rapid acceleration at commencement (Garland, 2005; Kleshnev, 2020a), high stroke rates, and an extended sprint phase. Predominant tactics employ a fast-start/fast-finish approach (Schünemann et al., 2023). Our prior research (Liu et al., 2021) segmented races into three phases: start, mid-race, and sprint. Notably, anaerobic metabolism dominates both the start and sprint segments, profoundly influencing overall outcomes. Consequently, the scientific assessment and development of rowers’ anaerobic capacity are critical for training optimization.

Traditional 2000-m ergometer tests, while ecologically valid, suffer from prolonged duration, cumulative fatigue, and limited sensitivity for quantifying short-term anaerobic power (Holmes et al., 2020). The Wingate test—predominantly lower-body-driven with fixed seating and minimal trunk engagement—underestimates rowing-specific anaerobic capacity due to significant biomechanical dissimilarities (de Ca et al., 2009). In contrast, the 30-s all-out rowing ergometer test provides superior specificity for evaluating explosive power and fatigue resistance, establishing it as a criterion method for assessing rowers’ anaerobic capabilities. Peak power output during this test explains 75.7% of the variance in race time, with the correlation between peak power output and race time being r = 0.90 (Cerasola et al., 2017). Additionally, VO_2_max and fatigue index contribute an additional 12.1% and 8.2%, respectively (Li, 2017).

Within the periodized annual training plan, the winter training block—defined here as a distinct phase dedicated to foundational physiological development—serves a unique and strategic role that justifies specific investigation. This phase is distinctly different from both the preparatory and competitive seasons in both its objectives and training structure. While pre-season and in-season training emphasize technical refinement, race-specific pacing, and performance maintenance, the winter phase is uniquely dedicated to foundational physiological development through a pronounced shift toward land-based conditioning. Training volume remains substantial but is reallocated, with a significantly increased emphasis on developing strength, power, and aerobic capacity in the gym and on ergometers, while on-water technical training is strategically maintained at a supplementary level to preserve skill proficiency. This concentrated focus on general physical qualities creates the primary annual window for inducing substantial physiological adaptations that form the basis for subsequent specialized performance.

The emphasis on developing strength and explosive power during winter training is predicated on their direct contribution to a rower’s ability to generate high force rapidly—a key determinant of performance in the start and sprint phases of a race, which are predominantly supported by anaerobic metabolism. Therefore, to holistically evaluate the efficacy of this distinct training phase, it is crucial to investigate not only the manifest improvements in physical performance but also the underlying metabolic adaptations within the anaerobic energy system. However, empirical evidence detailing these specific anaerobic metabolic adaptations in elite female rowers remains scarce, particularly concerning the responses to maximal, short-duration efforts. This study consequently aimed to examine whether a conventional winter training block enhances short-duration anaerobic metabolic output by comparing pre-versus post-training measures of mean power output, blood lactate accumulation, and anaerobic energy contribution during 30-s maximal rowing ergometer tests.

## 2 Methods

### 2.1 Participants

Five elite female rowers (age: 20.0 ± 2.5 years; body weight: 62.4 ± 1.8 kg; height: 173.2 ± 1.2 cm; BMI: 20.8 ± 0.3 kg/m^2^; training history: 4.8 ± 1.6 years) were recruited. The elite competitive status of all participants was rigorously defined and verified by their formal selection to the Chinese National Rowing Team training squad. Additional inclusion criteria were: freedom from musculoskeletal injuries for ≥6 months, regular menstrual cycles (25–35 days), no use of ergogenic supplements, and absence of cardiovascular contraindications. To control for potential hormonal influences on energy metabolism, all participants were tested during the early follicular phase (days 2–6) of their menstrual cycle for both pre- and post-training assessments. Written informed consent was obtained from all participants and/or their legal guardians after full disclosure of the procedures and risks. The protocols were approved by the Ethics Committee of Jiangxi Normal University (IRB-JXNU-PEC-20191110) in accordance with the Declaration of Helsinki.

### 2.2 Experimental design and setting

A repeated-measures design was implemented to assess physiological adaptations before (November 20–22, 2019) and after (March 23–25, 2020) a 16-week winter training period. Testing was conducted at the Hongfeng Lake Aquatic Training Base, Guizhou (altitude: 1,100 m) under controlled environmental conditions (temperature: 25.0 °C ± 0.5 °C; humidity: 66% ± 2%).

### 2.3 Winter training intervention

The 16-week periodized winter training program was designed in accordance with conventional practices for elite rowers. Its defining feature, compared to the subsequent pre-season and competitive seasons, was a strategic emphasis on high-volume, land-based physical conditioning aimed at developing a broad physiological foundation, with on-water technical training maintained at a supplementary level. This contrasts with later seasons where training focus shifts to race-specific pacing, technical refinement, and performance peaking.

The program comprised three sequential phases: 1. A 6-week Basic Preparation Phase emphasizing aerobic endurance (constituting ∼70% of the training volume) and foundational strength training (3 sessions/week). 2. A 5-week Intensification Phase focused on anaerobic threshold work (∼45% volume) and high-resistance rowing (∼15% volume). 3. A 5-week Specialization Phase prioritizing race-pace intervals (∼30% volume) and maximal power development. Training load was quantified using the session-RPE method (sRPE: 6,500 ± 500 AU).

### 2.4 Instrumentation

All tests utilized a CONCEPT2 rowing ergometer (Morrisville, VT, United States), with metabolic measurements collected via a Jaeger Oxycon Mobile portable gas analyzer (Vyaire, Höchberg, Germany). Blood lactate concentration was analyzed using a Biosen C-Line Clinic analyzer (EKF Diagnostics, Barleben, Germany) following a standardized protocol: 10 μL of capillary blood was drawn from sterilized fingertips, immediately transferred to 500 μL pre-loaded EKF Biosen C-Line reagent tubes, vortex-mixed for 1 min, incubated for 4 min at room temperature, and analyzed on the benchtop analyzer. Heart rate was monitored via Polar H10 sensors (Kempele, Finland). All equipment was used in accordance with the manufacturers’ guidelines and specifications.

### 2.5 Testing protocol

To minimize the influence of external factors on energy metabolism, participants were provided with standardized meals (comprising 65%–70% carbohydrate, 15%–20% fat, and 15%–20% protein) 2 h before all testing sessions. Participants also abstained from vigorous exercise (48 h) and caffeine/alcohol (24 h) prior to testing.

After anthropometric measurements, subjects completed two exercise tests on the same day, separated by a 10-min passive recovery period. This sequence was chosen to mirror the demands of a typical testing session in an elite training environment and to ensure the assessment of maximal anaerobic capacity in a non-fatigued state. The tests were conducted in the following order:

30-s all-out rowing Test: Following a 15-min standardized warm-up, participants performed a 30-s all-out rowing test. Power output was recorded at 1-s intervals. Capillary blood samples were collected at the 3rd, 5th, and 7th minute post-exercise, with the highest value recorded designated as the peak blood lactate concentration.

Incremental Rowing Test to Exhaustion: This test commenced at a workload of 2.0 W/kg body weight, with 30-W increases every 2 min until volitional exhaustion. Ventilatory parameters were recorded breath-by-breath.

**FIGURE 1:**
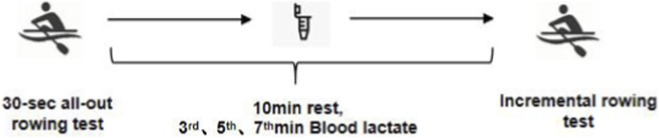
Flowchart of experimental design. Note: 30s all-out rowing test: anaerobic capacity test. Incremental Rowing Test (aerobic capacity test): Commenced at 2.0 × body weight (W), with 30W increases every 2 min. Test terminated at volitional exhaustion.

Standardized verbal encouragement was provided throughout to ensure maximal effort. The testing schedule is summarized in Figure 1.

### 2.6 Calculations

Anaerobic energy contribution was quantified via the maximal accumulated oxygen deficit (MAOD) method (Massini et al., 2023) using an incremental test protocol. Participants completed 6-8 discrete 2-min stages with progressively increasing intensity (±5% power output tolerance), continuing until: (a) failure to maintain target power, or (b) attainment of VO_2_max (confirmed by respiratory exchange ratio ≥1.10, oxygen uptake plateau [<150 mL min^-1^ increase], and heart rate ≥180 bpm).

For each participant, a subject-specific linear regression model was established between mean power output and oxygen uptake (measured during the final 30 s of each stage). This regression was used to calculate the theoretical oxygen demand (VO_2_demand) for the 30-s all-out trial. Actual oxygen uptake (VO_2_measured) during the maximal effort was recorded via gas analysis. The accumulated oxygen deficit (AOD) was computed as: AOD = Σ (VO_2_demand- VO_2_measured).

Aerobic energy contribution was derived from measured oxygen uptake (conversion: 21.131 kJ L^-1^ O_2_), while anaerobic contribution represented the temporal integration of oxygen deficit. Percentage contributions were calculated as:

Aerobic contribution (%) = (VO_2_measured/VO_2_demand) × 100;

Anaerobic contribution (%) = 100 - Aerobic contribution

**FIGURE 2:**
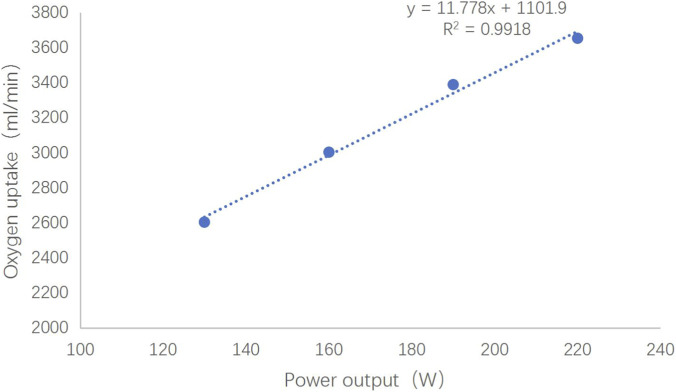
Subject-specific linear regression between power output and oxygen uptake during incremental rowing testing. Note: The established relationship (y = 11.778x + 1101.9; *R* ^2^ = 0.9918) was used to calculate theoretical oxygen demand (VO_2_ demand) for maximal accumulated oxygen deficit (MAOD) determination. Individual regression models were developed for all athletes (n = 5), with this participant exemplifying the methodological approach.

Energy utilization efficiency (η) was determined by:

$$\eta =\left[\text{Mechanical work output }\left(\text{kJ}\right)\;/\text{ Total energy expenditure }\left(\text{kJ}\right)\right]\times 100\%$$

where total energy expenditure = (VO_2_measured × 21.131) + Anaerobic energy equivalent. Figure 2 illustrates the regression methodology for a representative participant.

### 2.7 Statistical analysis

Data analysis was performed using SPSS 26.0 (IBM Corp., Armonk, NY, United States). Normality was assessed using Shapiro-Wilk tests. Parametric data are presented as mean ± standard deviation and analyzed using paired t-tests; non-parametric data were analyzed using Wilcoxon signed-rank tests. Statistical significance was set at p < 0.05 (two-tailed), with effect sizes reported as Cohen’s d for significant findings.

## 3 Results

### 3.1 Comparison of 30-s anaerobic capacity of rower pre- and post-winter training

The 30-s all-out rowing test results (Table 1) revealed significant post-winter training alterations: Absolute mean power output increased (t = 9.324, 95% CI [36.52, 67.48] w, P = 0.001), while body weight-normalized mean power output improved significantly (t = 8.580, 95% CI [0.71, 1.38] w, P = 0.001). Concurrently, post-exercise peak blood lactate concentration rose (t = 3.541, 95% CI [0.52, 4.29] mmol/L, P = 0.024), though oxygen uptake decreased (t = −3.018, 95% CI [-280.91, −11.73] mL, P = 0.039). No significant body mass changes were observed (t = −2.339, 95% CI [-4.156, 0.356] kg, P = 0.079).

**TABLE 1:** Comparison of 30-s anaerobic capacity of rowers pre- and post-winter training.

Stage	Boby weight (Kg)	Power output		VO_2_measured (mL)	Peak blood lactate (mol/L)
		MP (W)	MP (W/Kg)		
Pre-winter training	62.4 ± 1.8	367.60 ± 33.27	5.88 ± 0.41	1013.83 ± 118.34	7.16 ± 2.17
Post-winter raining	60.5 ± 2.8	419.6 ± 42.61^**^	6.93 ± 0.52^**^	867.51 ± 120.70^*^	9.56 ± 1.86^*^

Compare with pre-winter training, *P < 0.05, **P < 0.01.

### 3.2 Comparison of energy provision of rower pre- and post-winter training

Results from the incremental rowing test confirmed that maximal oxygen uptake (V̇O_2_max) did not change significantly following the training period (60.86 ± 6.16 V s. 59.53 ± 4.70 mL kg^-1^·min^-1^, p > 0.05). This ensured the validity of the subsequently calculated maximal accumulated oxygen deficit (MAOD). Table 2 indicates that winter training significantly increased total energy expenditure during 30-s all-out rowing in absolute terms (t = 3.461, 95% CI [1.35, 12.29] kJ, P = 0.026) and body weight-normalized values (t = 4.433, 95% CI [0.05, 0.23] kJ/kg, P = 0.011). Specifically, anaerobic expenditure demonstrated significant elevation in absolute output (t = 3.982, 95% CI [3.00, 16.82] kJ, P = 0.016), body weight-normalized output (t = 5.245, 95% CI [0.08, 0.27] kJ/kg, P = 0.006), and contribution (t = 3.775, 95% CI [2.51%, 16.47%], P = 0.020).

**TABLE 2:** Energy metabolism and energy efficiency within the 30-s all-out rowing.

Variables		Before	After
Total energy expenditure	Absolute (KJ)	56.53 ± 9.67	63.35 ± 10.34^*^
	Relative (KJ/kg)	0.91 ± 0.16	1.05 ± 0.16^*^
Aerobic expenditure	Absolute (KJ)	21.42 ± 2.50	18.33 ± 2.55^*^
	Relative (KJ/kg)	0.34 ± 0.32	0.30 ± 0.04
	contribution (%)	38.5 ± 5.7	29.1 ± 2.3^*^
Anaerobic expenditure	Absolute (KJ)	35.10 ± 8.92	45.02 ± 8.06^*^
	Relative (KJ/kg)	0.56 ± 0.15	0.74 ± 0.12^**^
	contribution (%)	61.5 ± 5.7	71.0 ± 2.3^*^
Energy efficiency	Contribution (%)	19.82 ± 2.67	20.10 ± 2.16

Compare with Pre-winter training, *P < 0.05, **P < 0.01.

Conversely, aerobic expenditure decreased significantly in absolute terms (t = −3.018, 95% CI [-5.94, −0.25] kJ, P = 0.039) and contribution (t = −3.894, 95% CI [-16.12%, −2.70%], P = 0.018), though no significant changes were observed in body mass-normalized aerobic output (t = −2.018, 95% CI [-0.09, 0.02] kJ/kg, P = 0.114) or energy efficiency (t = 0.619, 95% CI [-0.98%, 1.54%], P = 0.569).

## 4 Discussion

### 4.1 Analysis of 30-s anaerobic capacity of rower pre- and post-winter training

The development of strength and power qualities is paramount in rowing training, particularly for the initial race phase where athletes must rapidly generate maximal force to initiate oar strokes and establish boat momentum. This requires well-developed speed-strength and power endurance to ensure prompt water entry and forceful application through each stroke (Kleshnev, 2020b). Our findings demonstrate a significant 9.4% increase in absolute mean power (MP) during the 30-s all-out rowing test following winter training. When normalized to body weight, the improvement was even more pronounced (11.0%), indicating enhanced power generation capacity independent of the slight reduction in athlete body mass.

Post-exercise blood lactate concentration, measured at the third, fifth, and seventh minutes with the peak value used for analysis, showed a substantial 34% increase after training. According to the contemporary Lactate Shuttle theory (Brooks, 2018), lactate serves as an important energy substrate and signaling molecule rather than merely a metabolic waste product. The elevated peak lactate concentration observed following training likely reflects both an increased glycolytic flux supporting higher power outputs and potentially enhanced efficiency in lactate distribution and utilization via intramuscular and intermuscular shuttle mechanisms. This adaptation would facilitate lactate oxidation within oxidative muscle fibers (primarily Type I and IIa) and other tissues, supporting the elevated work rate (Fitts and Widrick, 1996).

Concurrently, we observed a significant 14.4% reduction in oxygen consumption during the test. This decreased aerobic demand at a substantially higher power output underscores the improved metabolic efficiency and anaerobic contribution following winter training.

### 4.2 Analysis of energy supply characteristics

The energetic profile of rowing has been extensively investigated since Hagerman’s seminal work (Hagerman et al., 1978). Li, (2017) examined energy system contributions across various durations of maximal rowing, demonstrating a shift from anaerobic to aerobic dominance as effort duration increases. Our study contributes to this literature by characterizing the energetic profile of a 30-s all-out rowing effort.

We employed the maximal accumulated oxygen deficit (MAOD) method to quantify energy system contributions. Our results indicate that winter training induced a significant shift toward greater anaerobic contribution (from 61.5% to 71.0%), with a corresponding decrease in aerobic contribution (from 38.5% to 29.1%). These findings align with Li Yongming’s report of 71.4% anaerobic contribution during a 45-s all-out effort (Li, 2017).

The observed 12.1% increase in total energy expenditure and 14.1% improvement in power output, coupled with the shift toward greater anaerobic metabolism, collectively indicate substantial enhancement of work capacity. From a metabolic perspective, the 28.3% increase in anaerobic contribution highlights improved anaerobic capacity and metabolic flexibility—critical attributes for sprint performance.

No significant change was observed in energy utilization efficiency. This suggests that while winter training effectively enhanced athletes’ physical capacities, it may not have substantially altered movement economy or muscle contractile efficiency in this short-duration task. Energy efficiency is influenced by multiple factors including muscle fiber type characteristics, neuromuscular coordination, and technical proficiency.

### 4.3 Potential mechanisms underlying performance enhancement

The significant improvements in mean power output and anaerobic energy contribution observed after winter training may be explained by several physiological adaptations documented in the literature. Although the present study did not directly measure morphological or molecular changes, the observed performance outcomes are consistent with well-established training-induced adaptations.

The increased anaerobic power output and elevated lactate concentration suggest an enhanced glycolytic flux. These findings align with potential mechanisms such as: (1) Fiber hypertrophy, particularly of fast-twitch fibers, which could increase the cross-sectional area for force production (Gehrig et al., 2010); (2) Upregulation and post-translational modifications of myosin light chains, which are known to improve cross-bridge cycling kinetics and power output (Borejdo et al., 2004); (3) Enhanced lactate shuttle efficiency, which would facilitate both intramuscular and intermuscular lactate distribution and oxidation (Li, 2017; Beneke et al., 2007).

Thus, the substantially elevated blood lactate concentration following training likely reflects both the increased glycolytic flux required to support higher power outputs and a potentially enhanced capacity for lactate clearance and utilization.

Collectively, these potential mechanisms provide a physiological framework for interpreting the observed performance enhancement, suggesting that winter training optimized both the contractile machinery and metabolic support systems to meet the demands of short-duration, maximal efforts.

### 4.4 Practical applications and perspectives

The performance enhancements observed in this study validate the physiological efficacy of conventional winter training periodization in rowing. Coaches and practitioners can be confident that the emphasis on strength and power development during this phase effectively targets the anaerobic qualities crucial for start and sprint performance. To optimize these adaptations, training programs should continue to incorporate exercises that elicit high glycolytic flux and challenge the neuromuscular system to produce force rapidly.

From a research perspective, while this study demonstrates the functional outcome of winter training, the underlying structural and molecular mechanisms warrant further investigation. Future studies employing muscle biopsies could directly examine training-induced changes in fiber type composition (specifically the IIa and IIx profiles), cross-sectional area, and the expression of proteins involved in the lactate shuttle (e.g., MCT1, MCT4). Additionally, integrating on-water measurements with portable metabolic systems would help bridge the gap between laboratory findings and actual racing performance.

## 5 Limitations

This study has several limitations that should be considered when interpreting the results. First, the sample size, though consisting of a homogeneous group of elite female rowers, was relatively small, which may affect the statistical power and generalizability of the findings. Second, the absence of body composition data, due to logistical constraints in transporting specialized equipment to the high-altitude training camp, limits our ability to determine whether the observed improvements were accompanied by changes in lean body mass. Third, while laboratory-based ergometer testing ensures standardized measurement of metabolic parameters, the fixed resistance profile of the rowing ergometer does not fully replicate the variable kinematics of on-water rowing. Fourth, the exclusive use of a 30-s all-out test, while optimal for assessing maximal anaerobic power and capacity, does not capture the metabolic responses and fatigue profiles associated with longer-duration, high-intensity efforts (e.g., 60–90 s) that are also critical to rowing performance.

## 6 Conclusion

In conclusion, a 16-week conventional winter training period significantly enhanced anaerobic performance in elite female rowers, as evidenced by increased mean power output, elevated post-exercise blood lactate concentration, and a greater reliance on anaerobic energy pathways during a 30-s all-out rowing test. These findings provide empirical physiological validation for current periodization practices in competitive rowing, confirming that winter training effectively develops the key physical qualities underpinning success in the critical start and sprint phases of a race.

## Data Availability

The raw data supporting the conclusions of this article will be made available by the authors, without undue reservation.
